# Risk Assessment of Fluxametamide Resistance and Fitness Costs in Fall Armyworm (*Spodoptera frugiperda*)

**DOI:** 10.3390/toxics11040307

**Published:** 2023-03-26

**Authors:** Debashis Roy, Sujan Biswas, Sukamal Sarkar, Samrat Adhikary, Gautam Chakraborty, Pijush Kanti Sarkar, Laila A. Al-Shuraym, Samy Sayed, Ahmed Gaber, Akbar Hossain

**Affiliations:** 1 Dhaanya Ganga Krishi Vigyan Kendra, Ramakrishna Mission Vivekananda Educational and Research Institute, Sargachhi, Murshidabad 742408, West Bengal, India; 2 School of Agriculture and Rural Development, Ramakrishna Mission Vivekananda Educational and Research Institute, Kolkata 700103, West Bengal, India; 3 Department of Agricultural Entomology, Bidhan Chandra Krishi Viswavidyalaya, Mohanpur, Nadia 741252, West Bengal, India; 4 Department of Biology, College of Science, Princess Nourah Bint Abdulrahman University, P.O. Box 84428, Riyadh 11671, Saudi Arabia; 5 Department of Economic Entomology and Pesticides, Faculty of Agriculture, Cairo University, Giza 12613, Egypt; 6 Department of Science and Technology, University College-Ranyah, Taif University, P.O. Box 11099, Taif 21944, Saudi Arabia; 7 Department of Genetics, Faculty of Agriculture, Cairo University, Giza 12613, Egypt; 8 Department of Biology, College of Science, Taif University, P.O. Box 11099, Taif 21944, Saudi Arabia; 9 Division of Soil Science, Bangladesh Wheat and Maize Research Institute, Dinajpur 5200, Bangladesh

**Keywords:** isoxazoline, fall armyworm, realized heritability, cross-resistance, detoxification enzyme, demography, life table

## Abstract

The fall armyworm (FAW), *Spodoptera frugiperda*, is one of the most devastating invasive polyphagous pests, which has attracted recent global attention by developing resistance to various insecticidal active ingredients with independent mode of action. Fluxametamide, a newly commercialized isoxazoline insecticide, is exceptionally selective towards several lepidopteran pests. The present study aimed to evaluate resistance risk in FAW to fluxametamide and the fitness costs associated with fluxametamide resistance. A field-collected and genetically mixed population of FAW was artificially selected through continuous exposure to fluxametamide. After successive selection of 10 generations, there was no obvious increase in the LC_50_ (RF: 2.63-fold). The realized heritability (*h*^2^) of fluxametamide resistance was estimated as *h*^2^ = 0.084 using a quantitative genetic approach. Compared with the susceptible F0 strain, the Flux-SEL (F10) strain of FAW displayed no significant cross-resistance to broflanilide, chlorantraniliprole, fipronil, indoxacarb, lambda cyhalothrin, spinetoram, and tetraniliprole, except emamectin benzoate (RF: 2.08-fold). Increased activity of glutathione *S*-transferase (ratio 1.94) was observed in the Flux-SEL (F10) strain of FAW, while the cytochrome P450 and carboxylesterase activities were not altered. The fluxametamide-selection significantly affected the development and reproductive traits of FAW with a lower *R_0_*, *T* and relative fitness (*R_f_* = 0.353). The results alluded that the risk of fluxametamide resistance evolution in FAW is relatively lower; however, proactive implementation of resistance management approaches should be done to maintain the field efficacy of fluxametamide against FAW.

## 1. Introduction

The fall armyworm (FAW), *Spodoptera frugiperda* (Lepidoptera: Noctuidae), is an agricultural pest that poses a danger to crop output globally due to its outstanding adaptability and high migratory and polyphagous behaviour [1,2,3,4,5]. By May 2018, researchers had found this insect pest for the first time in southern India [6]. Since then, it has quickly spread to numerous other Asian countries, severely affecting local crops such as maize [7,8,9,10]. The meteorological, biological, and agronomic variety of the Indian subcontinent is extremely different [11], which is expected to have an impact on the dynamics of the FAW population and its management. Even though native biological control agents with the potential to manage FAW, such as natural predators, parasitoids, and entomopathogenic fungi, have recently been shown to be effective [12,13,14], the agricultural department of the Indian government still advises using synthetic insecticides for chemical control in cases of severe FAW infestations [15]. However, the FAW has developed resistance to almost all classes of conventional and novel insecticides, including organophosphates, carbamates, synthetic pyrethroids, fiprols, diamides, spinosyns, benzoylureas [16,17,18,19,20], and *Bacillus thuringiensis* Cry toxins as a result of extensive field application [21,22,23,24]. New active ingredients with novel target sites are therefore required for the successful control of FAW.

Unlike diamides, avermectins, and fiproles, fluxametamide is a next-generation reduced-risk isoxazoline insecticide. It is categorised in group 30 of the Insecticide Resistance Action Committee mode of action (MoA) classification [25] and has a new target site for insect pests that interferes with GABA Cl^−^ and Glu Cl^−^ channels [26,27]. Fluxametamide has exceptional insecticidal activity against a variety of insect orders, including Lepidoptera, Thysanoptera, Coleoptera, and Diptera [28]. It also has the advantage of having very low non-target toxicity, which benefits the prevailing natural enemies and insect pollinators [26,29]. Fluxametamide is effective against fipronil-resistant pest populations due to its distinct binding location in GABA-gated chloride channels, which is different from those for other antagonists [30]. Fluxametamide, which was previously introduced in China, Australia, and Japan, was recently introduced in India and is anticipated to be used successfully to control a variety of lepidopteran insects [31,32]. However, the resistance of FAW, one of the most important invasive insect pests, to fluxametamide is still unexplored.

Before a thorough field application of a reduced-risk pesticide, it is crucial to quantify the resistance risk for a certain insect pest. Formulating resistance management techniques requires information from studies of insecticide resistance, as well as data on realized heritability and related traits from insecticide selection tests [33,34]. Fluxametamide, a new-generation reduced-risk insecticide of the 21st century, has been commercialized recently in the global market, and very little information is available on its toxicity and resistance-related parameters on various insect pests. Sublethal effects of fluxametamide on *Plutella xylostella* [31] and *Chilo suppressalis* [35] have been reported recently by previous workers, and the resistance risk assessment of this insecticide in *P. xylostella* was also studied [32]. Therefore, to restore the susceptibility for a longer period and to formulate proper management strategies of fluxametamide resistance against one of the most important invasive insect pests, FAW, information on the resistance risk of fluxametamide and fitness costs in FAW should be generated. In the present study, the risk of FAW gaining resistance to fluxametamide was determined after laboratory-selected resistance and cross-resistance to other widely used insecticides were evaluated. In addition, the relative resistance and susceptibility of the fluxametamide-selected (Flux-SEL) generations (F5 and F10) were examined in terms of changes in the activities of cytochrome P450, glutathione *S*-transferase, and carboxylesterase. The findings inform future research on the molecular mechanisms behind the development of fluxametamide resistance in FAW and offer scientific advice for the use of fluxametamide and the control of resistance in this invasive insect pest.

## 2. Materials and Methods

### 2.1. Insect Populations and Rearing 

In the year 2020, a total of six field populations of *S. frugiperda* larvae were collected from conventional commercial maize fields in four different provinces in India (Table S1) without any prior history of insecticide resistance [9,36]. Insect larvae were fed with an artificial corn-based diet [37] and individual populations were reared under controlled climate conditions at 25 ± 2 °C temperature, 70 ± 5% relative humidity, and a photoperiod of 16 light: 8 dark [38]. After maintaining the field populations for a single generation in the laboratory, 40 mated females from each of the six strains were used to generate a mixed population to have maximum genetic diversity. Larvae of the first generation from the mixed population were regarded as the susceptible (F0) strain of FAW and utilized in the bioassay for the selection of fluxametamide resistance in the laboratory. The diet was replaced as and when required. The pupae were collected, weighed individually, and placed in an adult emergence chamber in darkness. The male and female moths were paired and shifted in plastic screen cages (35 cm × 35 cm × 45 cm, L × B × H) for mating and oviposition. Adults were fed with 10% *w*/*v* fresh honey solution.

### 2.2. Insecticides 

For the resistance selection bioassay, a formulated market product of fluxametamide 100 g a.i. L^−1^ (Gracia^®^ 10% *w*/*w* EC, Godrej Agrovet, Mumbai, India) was used. In addition, commercial formulations of broflanilide 300 g a.i. L^−1^ (Exponus^®^ 300 G/L SC, BASF, India), chlorantraniliprole 185 g a.i. L^−1^ (Coragen^®^ 18.5% *w*/*w* SC, FMC Corporation, Maharashtra, India), emamectin benzoate 50 g a.i. KG^−1^ (Proclaim^®^ 5% SG, Syngenta, Tamil Nadu, India), fipronil 800 g a.i. KG^−1^ (Jump^®^ 80% *w*/*w* WG, Bayer Crop Science, Thane, India), indoxacarb 145 g a.i. L^−1^ (King Carb^®^ 14.5% SC, Parijat Industries Ltd., Delhi, India), lambda cyhalothrin 49 g a.i. L^−1^ (Lamcy^®^ 4.9% CS, R K Chemicals, Gujarat, India), spinetoram 117 g a.i. L^−1^ (Largo^®^ 11.7% SC, Dhanuka Agritech Ltd., Haryana, India), and tetraniliprole 200 g a.i. L^−1^ (Vayego^®^ 200 SC, Bayer Crop Science, Karnataka, India) were used for the cross-resistance bioassays.

### 2.3. Bioassays

The IRAC-recommended ‘diet incorporation assay’ [39] with slight modifications was used to conduct the bioassays with different insecticides. In brief, third instar FAW larvae were individually fed into 1 mL of artificial corn-based food fully mixed with a series of pesticide dilutions encompassing 0 to >98% mortality in each petri dish (diameter 3.5 cm) and covered with a nylon mesh net. Bioassays consisted of seven different concentrations for each insecticide (0.00, 0.01, 0.05, 0.10, 1.00, 5.00, and 10.00 mg L^−1^), including control (diet mixed with double-distilled water only), and were replicated four times. Thus, 280 numbers of FAW third-instar larvae were used in each insecticide bioassay, maintained under the aforementioned controlled climate conditions. Larval mortality was recorded after 96 h. Larvae that did not respond after probing with a camel-hair brush or individuals whose growth was reduced by approximately 30% of the size of the control treatment were considered to be dead.

### 2.4. Selection Using Fluxametamide

A group of 280 third-instar larvae was selected from the first generation of the laboratory-generated mixed population of FAW as susceptible (F0) strain, and exposed to fluxametamide for 10 successive generations. Each succeeding generation was selected using the upper quartile LC_75_ (0.031–0.094 mg L^−1^) concentration of fluxametamide based on the bioassay data of the preceding generation. After 96 h of fluxametamide exposure, the larval mortality was recorded, and the surviving FAW larvae were allowed to breed for the next generation by transferring onto the fresh corn-based diet. The bioassay for fluxametamide-resistance selection followed by the rearing of survivors was performed under the previously mentioned controlled laboratory conditions.

### 2.5. Estimation of Realized Heritability (h^2^)

The realized heritability (*h*^2^) of fluxametamide resistance in FAW was estimated using a threshold trait analysis method [40,41]:

(1) $$h^{2}=R/S$$

The terms “selection response” (*R*) and “selection differential” (*S*) refer, respectively, to the “difference in average phenotype between the progeny of the selected parents and the parental generation before selection” and “difference in average phenotype between the selected parents and the entire parental generation”. The calculation of *R* and *S* has been given in our previous publication [32]. Assessment of fluxametamide resistance risk was done using the theory of Tabashnik [40]. The value of *h*^2^ (either greater or less)^2^ was used to calculate the slope of the predicted rate of resistance evolution. When LC_50_ (G) increased by 10 times at a selection intensity of 50–95%, the number of generations was enumerated. The resistance development rate was anticipated from the realized heritability (*h*^2^) and mortality rate.

(2) $$G=1/R=1/h^{2}S$$

### 2.6. Detoxification Enzyme Activities

The activity of cytochrome P450 was measured according to Chen et al. [42] with slight modifications. The NADPH was used as a substrate and the reaction was done in a MicroAmp 96-well plate (Thermo Scientific, Waltham, MA, USA) using 100 μL of 2 mM p-nitroanisole solution in each well. Each reaction mixture consisted of 90 μL of crude enzyme source, incubated at 27 °C for 3 min, and 10 μL of 9.6 mM NADPH pH 7.6 was added to make a final volume of 100 μL. The mixture without the enzyme source was regarded as a control. All reactions were replicated eight times and measured using a microplate reader (Agilent BioTek 800 TS, Marshfield, WI, USA). The P450 activity was enumerated as pmol min^−1^ mg protein^−1^. The protein concentration in the enzyme source was determined using the method of Bradford [43].

Using CDNB and GSH as substrates, the glutathione *S*-transferase (GST) activity was accessed by adapting MicroAmp 96-well plate following Nauen and Stumpf [44] with slight modifications. A 25 μL quantity of substrate solution (0.05 M HEPES buffer pH 6.8 with 0.1% (*v/v*) Tween-80; 0.4 mM CDNB and 4 mM GSH at the final concentrations) and 25 μL of enzyme source were used in the reactions. The reaction mixture without the enzyme solution was treated as a control. All reactions were replicated eight times, and continuously for 5 min, the change in absorbance was measured at 340 nm wavelength and 25 °C, using a spectrofluorometer (Edinburgh Instruments Ltd., Livingston, UK).

The activity of carboxylesterase was determined by following the methods of Grant et al. [45] with few modifications. The substance solution (0.1 mL 100 mM in Alpha-NA, 10 mg Fast Blue RR salt, and 5 mL 0.2 M sodium phosphate buffer of pH 6.0) of 90 μL and enzyme solution of 10 μL were mixed and poured in each well of a MicroAmp 96 well plate (Thermo Scientific USA). The wells with substrate solution instead of enzyme source served as control and each reaction was replicated eight times. Absorbance data were continuously recorded at 450 nm and 25 °C for 10 min in every 1.5 min interval using a spectrophotometer (Shimadzu, UV-1900). The quantity of the enzyme activity was estimated as nmol min^−1^ mg protein^−1^.

### 2.7. Evaluation of Fitness Costs

To construct the separate life tables of susceptible F0, and fluxametamide-selected F5 and F10 generations of FAW, 100 numbers of eggs in each generation were used. Besides, another set of 10 egg masses (more than 100 eggs per mass) in each generation of F0, F5, and F10 was also collected on the 3rd day of the egg-laying period for adult female moths to study the egg hatching rate. For the life table, newly hatched larvae were individually placed into a plastic tube (diameter 2 cm; height 8 cm), containing a fresh artificial corn-based diet, using a soft camel-hair brush. The diet was replaced as and when required. Data were recorded on the developmental duration of larval instars and following stages, pre-oviposition and oviposition duration, pupation rate, and pupal weight. After adult emergence, male and female moths were paired and placed into the previously mentioned plastic screen cages for oviposition. The survival rate of adults and fecundity of female moths were also recorded.

### 2.8. Data Analysis

Then, necessary bioassay results were adjusted for control mortality using Abbott’s formula [46]. For the Probit analysis, data were processed using PoloPlus software 2.0 (Leora, MO, USA). Any two LC_50_ values were determined to be significantly different when their 95% confidence intervals of the resistance factor did not include 1 [47]. Using SPSS software (Version 18.0: Inc., Chicago, IL, USA), the development and reproductive parameters, as well as the activities of each metabolic enzyme, were compared across the tested generations of FAW using a one-way ANOVA followed by Tukey’s HSD tests. The life table parameters at age *x* and stage *j*, such as age-specific survival rate (*l_x_*), age-stage specific survival rate (*s_xj_*), age-specific maternity (*l_x_m_x_*), age-specific fecundity (*m_x_*), age-stage specific life expectancy (*e_xj_*), mean generation time (*T*), net reprobation rate (*R_0_*), intrinsic rate of increase (*λ*), and finite rate of increase (*r*) were analyzed based on the theory of the age-stage and two-sex life table [48,49] using TWOSEX-MS chart program [50]. The mean values and the standard errors (SEs) of the life table parameters were obtained using a non-parametric method employing 10,000 bootstrap replicates. The paired bootstrap test (*p* < 0.05) was done using the TWOSEX-MS chart program to calculate the significant differences across the three tested generations of FAW.

## 3. Results

### 3.1. Assortment for Fluxametamide Resistance

Table 1 shows the response of FAW to fluxametamide selection over 10 consecutive generations. The LC_50_ values of fluxametamide for third FAW instars after 10 generations of selection were 0.024–0.063 mg L^−1^. Fluctuation of susceptibility was observed in different fluxametamide-selected generations of FAW, and the final resistance factor (RF) for F10 generation was 2.63-fold.

### 3.2. Estimation of Fluxametamide Resistance Development

**Figure 1:**
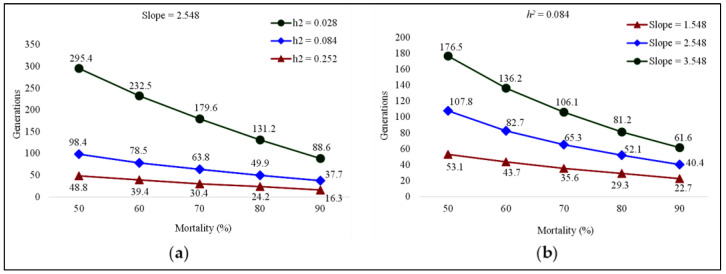
Effect of realized heritability (*h*^2^) (**a**) and slope (**b**) on the number of generations of FAW required for a 10-fold increase in LC_50_ of fluxametamide at different selection intensities (*i*).

The realized heritability (*h*^2^) of fluxametamide resistance in FAW was 0.084, with a mean *S* of 0.501 and a mean response *R* of 0.042 (Table 2). The predicted rate of fluxametamide resistance evolution is directly proportional to *h*^2^ and selection intensity (Figure 1a), but inversely proportional to the slope (Figure 1b). At the slope of 2.548, if the *h*^2^ is 0.084, then 63.8–98.4 generations are needed to obtain a 10-fold increase in resistance at 50–70% selection mortality. Similarly, at 90% mortality, when the *h*^2^ is 0.028 and 0.252, the resistance factors would increase by 10-fold after 88.6 and 16.3 generations, respectively. In contrast, at a constant *h*^2^ of 0.084, when the slope value is 1.548, a 10-fold increase in fluxametamide resistance will have occurred after 53.1 generations of FAW at a mortality of 50%. Likewise, at the slope of 3.548, and the selection intensity of 70–90%, 61.6–106.1 generations are required to increase resistance by a factor of 10.

### 3.3. Cross-Resistance Pattern

Compared with the susceptible (F0) population of FAW, the fluxametamide-selected (Flux-SEL) F10 generation showed no significant cross-resistance to broflanilide (1.16-fold), chlorantraniliprole (1.43-fold), fipronil (1.31-fold), indoxacarb (1.18-fold), lambda cyhalothrin (1.24-fold), spinetoram (1.05-fold), and tetraniliprole (1.39-fold) (Table 3). However, a lower level of cross-resistance in the Flux-SEL (F10) strain of FAW was observed to emamectin benzoate (2.08-fold).

### 3.4. Activities of Detoxification Enzymes

The major detoxification enzyme activities such as cytochrome P450, glutathione *S*-transferase, and carboxylesterase were assessed at an interval of five generations of FAW during the selection process using fluxametamide (Table 4). Compared to the susceptible (F0) population, the activity of P450 was significantly higher (elevated 1.13-fold) in Flux-SEL (F10) generation, but the Flux-SEL (F5) generation did not differ significantly. Furthermore, a significant increase in GST activity was observed in both the Flux-SEL (F5 and F10) populations of FAW with an elevated 1.21-fold and 1.94-fold, respectively. However, no significant difference was observed in the activity of carboxylesterase (*p* = 0.092) among the tested generations of FAW selected with fluxametamide.

### 3.5. Effects of Fluxametamide Selection on the Growth and Reproductive Parameters of FAW

The effects of fluxametamide selection on the developmental duration of FAW are depicted in Table 5. 

The Flux-SEL (F10) generation significantly prolonged the first and fifth instar larval duration by 0.16 (*p* < 0.0001) and 0.18 (*p* < 0.0001) days, respectively. Similarly, the sixth larval and pupal duration were also significantly longer in both Flux-SEL (F5) and Flux-SEL (F10) generations by 0.28 and 0.82 days (*p* = 0.019), and 1.14 and 1.83 days (*p* < 0.0001), respectively. However, the longevity of adult females (*p* = 0.0037) was significantly reduced by 0.33 and 0.77 days in the fluxametamide-selected F5 and F10 generations, respectively. No significant difference (*p* > 0.05) was observed between the flux-SEL (F5 and F10) and susceptible (F0) generations concerning the developmental duration of egg, second, third or fourth larval instars, or adult male longevity. 

Regarding the reproductive traits, the oviposition period was significantly increased (*p* < 0.0001) by 1.78 days in flux SEL (F10) generation (Table 6). Furthermore, the pupation rate was significantly decreased (*p* = 0.0052) in Flux-SEL (F5 and F10) generations by 7.18% and 23.89%, respectively. In addition, a significant diminution (*p* < 0.0001) was observed in pupal weight (0.13 g) of FAW selected with fluxametamide after a successive 10 generations. However, fluxametamide selection did not pose any significant difference in the pre-oviposition period or fecundity or hatchability of FAW.

### 3.6. Effects of Fluxametamide Selection on the Fitness Costs and Life Table Parameters of FAW

**Figure 2:**
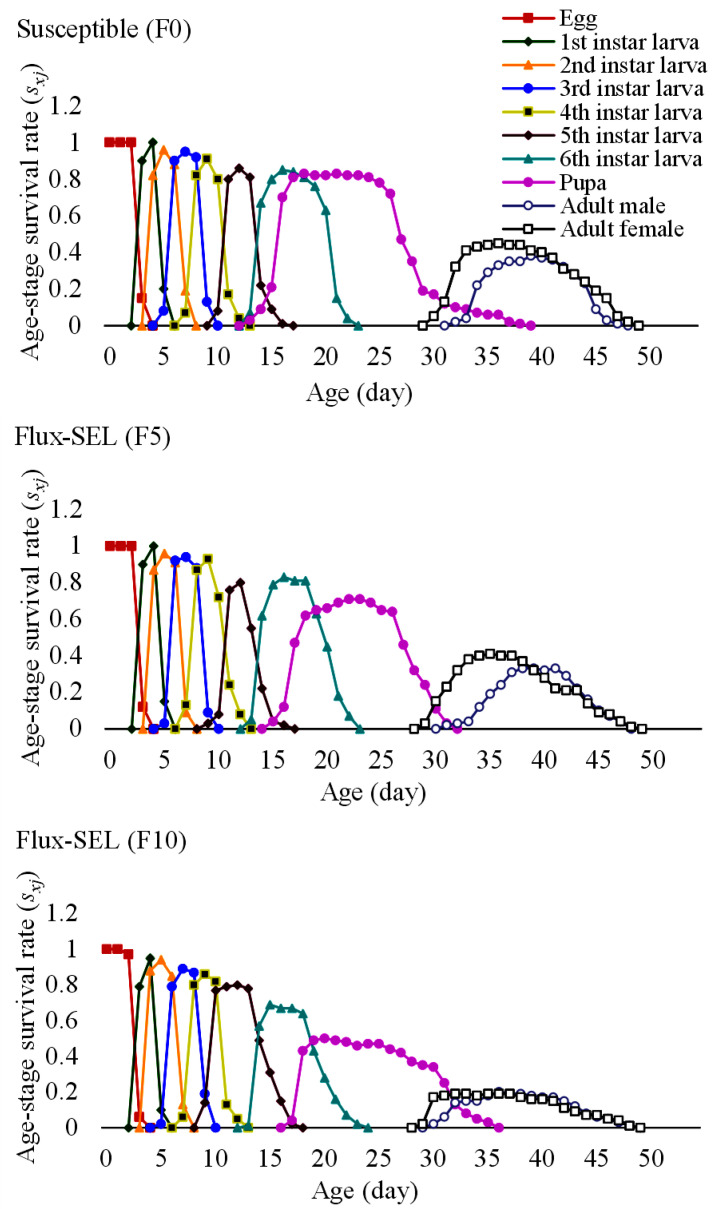
Age-stage specific survival rate (*s_xj_*) of FAW in F0, F5, and F10 generations selected with fluxametamide.

The age-stage-specific survival rate (*s_xj_*) of FAW showed a clear overlap between the fluxametamide-selected (F5 and F10) generations and susceptible (F0) generation (Figure 2). 

**Figure 3:**
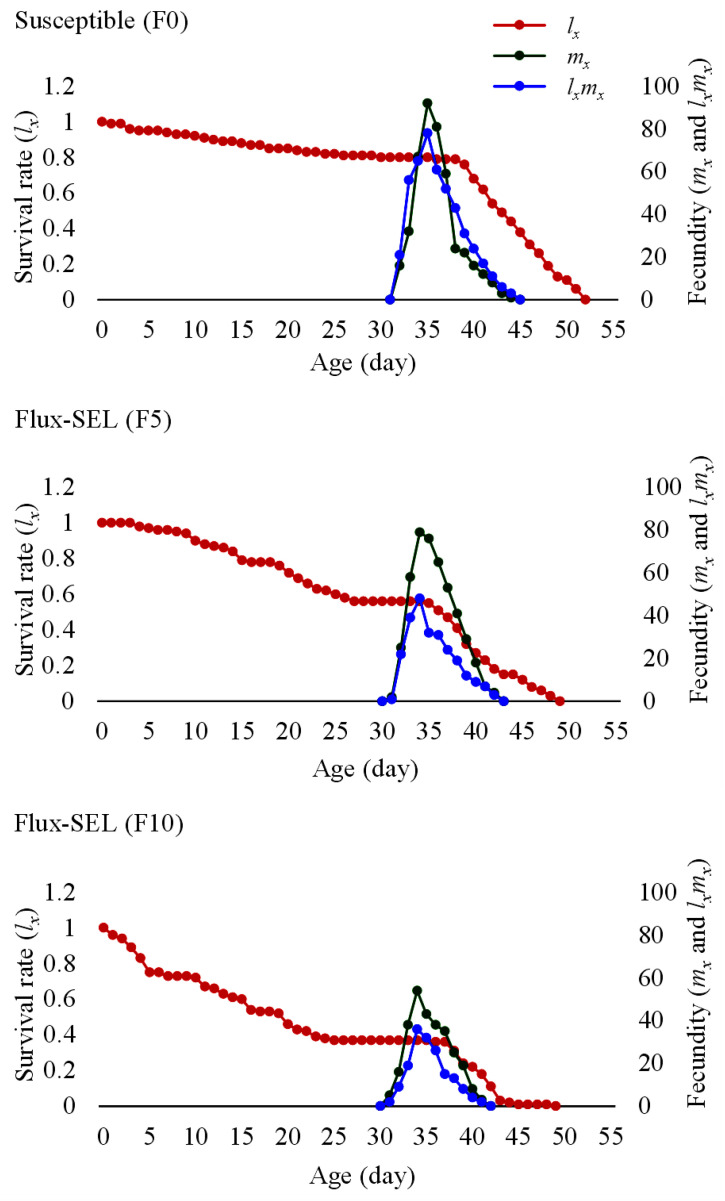
Age-specific survival rate (*l_x_*), fecundity (*m_x_*), and maternity (*l_x_m_x_*) of FAW in F0, F5, and F10 generations selected with fluxametamide.

**Figure 4:**
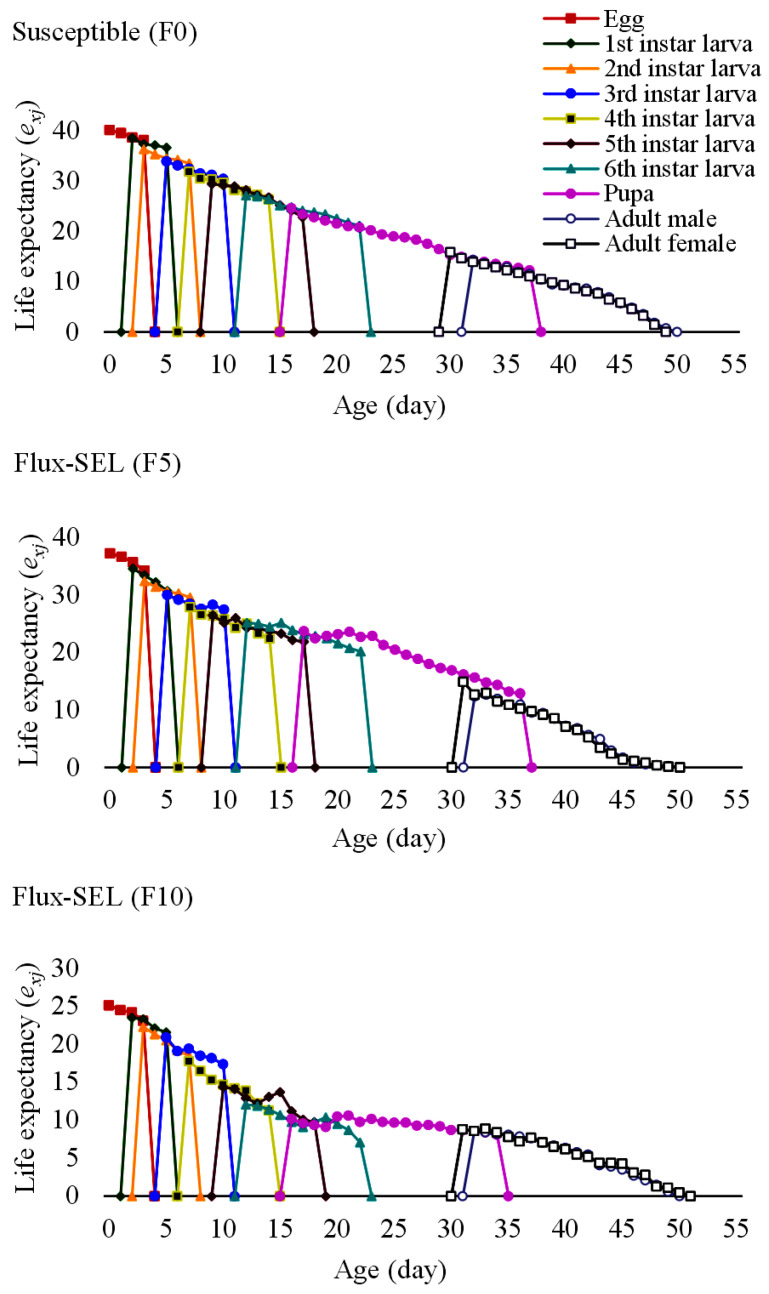
Age-stage specific life expectancy (*e_xj_*) of FAW in F0, F5, and F10 generations selected with fluxametamide.

A lower survival duration of 49 days for both Flux-SEL (F5 and F10) generations than the susceptible (F0) generation (52 days) was observed from the age-specific survival rate (*l_x_*) curve (Figure 3). Similarly, a decreasing trend as susceptible (F0) > Flux-SEL (F5) > Flux-SEL (F10) was also encountered from the age-specific fecundity (*m_x_*) and age-specific maternity (*l_x_m_x_*). Irrespective of the tested populations, a reduced life span with the advancement of age was noticed from the life expectancy (*e_xj_*) curves of FAW with a longer life in fluxametamide-selected (Flux-SEL) strain (F10) compared to the F0 strain (Figure 4). The egg stage exhibited the maximum *e_xj_* of 40.1, 37.1, and 25.2 days in F0, Flux-SEL (F5), and Flux-SEL (F10) generations, respectively. 

The life table components of FAW were compared between the susceptible (F0) and Flux-SEL (F5 and F10) generations (Table 7). The net reproductive rate (*R_0_*) (*p* = 0.0014) and the mean generation time (*T*) (*p* = 0.0125) were significantly decreased for Flux-SEL (F10) generation (257.14 offspring/individual and 31.712 days, respectively) compared to susceptible (F0) generation (728.24 offspring/individual and 35.434 days, respectively). However, the intrinsic rate of increase (*r*) and finite rate of increase (*λ*) did not differ significantly after fluxametamide selection in FAW. The calculated *R_f_* values for fluxametamide-selected F5 and F10 generations were 0.741 and 0.353, respectively.

## 4. Discussion

The best method for controlling FAW is still thought to be spraying with chemical or biologically originated insecticides [9]. Unfortunately, the field efficacies of many traditional and new chemical insecticides have been drastically reduced worldwide due to the rapid evolution of insecticide resistance to FAW [17,19,51,52,53]. To establish appropriate application instructions for a new active compound commercialized in the global market, it is critical to comprehend insecticide resistance in the target insect pests [54]. Fluxametamide, a brand-new isoxazoline insecticide, is highly efficient against a variety of lepidopteran, thysanopteran, coleopteran, and dipteran pests [28] by affecting insect GABA-gated chloride channels (GABA Cls). This molecule has gained increased attention recently as a member of a unique chemical class that may successfully manage fipronil-resistant insect pests [26].

Bioassay results of the present study indicated that the susceptibility of FAW to fluxametamide significantly varied among the selected generations with the increase in insecticide concentrations used throughout the selection experiment. After the fluxametamide selection for consecutive 10 generations, the Flux-SEL (F10) strain of FAW exhibited a low level of resistance factor (2.63-fold) compared to the susceptible (F0) population. The active ingredient, the baseline resistance frequency, and the biological characteristics of the pest, as well as the insecticide selection pressure, are all directly related to the pace of insecticide resistance development [55,56]. The delayed rate of resistance evolution in FAW during the fluxametamide selection may be connected to the poor primary frequency of insecticide resistance in the tested population. However, in terms of insecticide resistance, FAW is currently considered one of the fifteen invasive insect pest species worldwide [52]. Therefore, to reduce the chance of insecticide resistance development in the control of FAW, proper precautions should be made in the selection and field application of insecticides.

Using the estimations of selection strength and the resistance development rate, the estimation of realized heritability of resistance offers a systematic method for listing the outcomes of selection experiments [34,57]. Additionally, the current analysis places the results of the selection experiment in the broader context of the theoretical and experimental evolutionary biology literature [58]. Our results indicated that the predicted realized heritability (*h*^2^) for FAW (*h*^2^ = 0.084) after 10 consecutive generations of selection with fluxametamide were lower than lambda cyhalothrin (*h*^2^ = 0.420), and methomyl (*h*^2^ = 0.140) [59], and also lower than *Spodoptera exigua* (*h*^2^ = 0.108) and *Plutella xylostella* (*h*^2^ = 0.180) selected with chlorantraniliprole [34] and fluxametamide [32], respectively. These observations of the low *h*^2^ value of FAW after fluxametamide selection might be attributable to the stronger involvement of environmental variation than the alleles of additive genetic variation present in FAW responsible for fluxametamide resistance. These hypotheses are in agreement with the studies of Roy et al. [32], Lai and Su [34], Ijaz et al. [55], and Abbas and Shad [57]. The resistance development rate was elicited using the slope values of the Probit lines in different selected generations and the estimated realized heritability. The predicted rate of fluxametamide resistance development in FAW is directly proportional to *h*^2^ but inversely proportional to the slope. Practically, the open-field condition differs from the controlled laboratory environment in several parameters, including pest population migration, the fluctuation of abiotic weather factors, and non-scientific insecticide application practices, such as rotation and tank-mixing of similar molecules, play a significant role in determining resistance [34,56]. Therefore, the potential risk of field-evolved resistance could be different than the resistance predicted in the laboratory. Although from the present study, it was quite clear that the FAW population with a diverse genetic background developed fluxametamide resistance very slowly and registered a low *h*^2^ value in the laboratory, further study on the baseline susceptibility and field-resistance monitoring of FAW towards fluxametamide should be carried out.

Revealing the cross-resistance pattern between two independent insecticidal active ingredients provides valuable information to formulate insecticide resistance management strategies [60]. In the present study, the Flux-SEL (F10) generation of FAW exhibited no significant cross-resistance to various insecticide formulations, *viz*., broflanilide, chlorantraniliprole, fipronil, indoxacarb, lambda-cyhalothrin, spinetoram, and tetraniliprole; however, a lower but significant cross-resistance to emamectin benzoate, a glutamate-gated Cl channel allosteric modulator insecticide, was observed. It might be attributed to the fact that fluxametamide has shown an asymmetric nature of cross-resistance with emamectin benzoate, which is an example of a sudden unpredictable phenomenon [61,62]. Several cases of cross-resistance between Cry1 proteins [63], spinosad and spinetoram [64], indoxacarb, and deltamethrin [65] in FAW have already been reported previously. Roy et al. [32] investigated a similar type of cross-resistance pattern between fluxametamide and emamectin benzoate in *P. xylostella*, another invasive lepidopteran insect pest. Furthermore, an asymmetric nature of cross-resistance between broflanilide (a novel meta-diamide insecticide belonging to the same group of fluxametamide in IRAC MoA classification) and abamectin (another member of avermectin compounds) was also observed in *P. xylostella* [66]. Based on the results of the current experiment and those of other researchers, we hypothesize that there may be a potential of cross-resistance between avermectin molecules and isoxazoline compounds. However, additional research will be required to resolve this discrepancy.

When a single molecule chooses an isoenzyme that can interact with other compounds, cross-resistance to insecticides with independent modes of action may be conceivable [67]. Detoxification enzymes, including cytochrome P450s, GST, and esterase genes, have been demonstrated to promote the capacity of FAW to metabolically detoxify a wide range of chemical insecticides [24,65,68]. In the present study, a gradual increase in the activities of P450 and GST was observed after the continuous exposure of fluxametamide to FAW, and was found to be significantly different at Flux-SEL (F10) generation. The findings indicate that GST and P450 possibly contribute to the decreased fluxametamide sensitivity of FAW. Similarly, elevated activity of GST was observed in the fluxametamide-selected populations of *P. xylostella* [32] and indicated that GST has a major role in the detoxification process of fluxametamide in various lepidopteran insect pests. Moreover, an alike detoxification mechanism of emamectin benzoate by increasing the GST activity was reported by Pu et al. [69] and Gong et al. [70]. It could be attributed to the fact that the potential cross-resistance between fluxametamide and emamectin benzoate in the fluxametamide-selected strain of FAW was a possible resultant effect of a similar detoxification mechanism concerning elevated GST activities. The involvement of P450 in the indoxacarb resistance [65] and GST in the resistance to synthetic pyrethroid insecticides [71] in FAW have been reported by previous workers. In this study, the activities of detoxification enzymes were mild in fluxametamide-selected generations of FAW; we speculate that the limited resistance development rate is correlated with the slow metabolic detoxification process. However, further characterization of fluxametamide resistance and the development of more effective insecticide resistance control methods for FAW require digital gene expression by transcriptome in subsequent molecular investigations.

To encounter insecticide resistance, a thorough investigation of the fitness costs and life table parameters of an insect pest exposed to an insecticide is essential [72,73]. On the onset of insecticide resistance evolution, FAW experiences an obvious fitness cost [65,74,75]. In the present study, even though FAW exhibited a low level of fluxametamide resistance after continuous exposure for 10 successive generations, a significant prolongation of the first, fifth, and sixth larval instar, and pupal duration were observed in the Flux-SEL (F10) generation. A significant increase in the larval duration of *P. xylostella* [31] and *C. suppressalis* [35] at the sublethal exposure of fluxametamide has already been reported previously. A lower appetite, feeding disturbance, an aberrant metabolism, starving stress, or an imbalance between physiological development and metabolic detoxification could all be reasons for the prolonged larval duration in fluxametamide exposure [76,77]. It is crucial to remember that the prolonged larval period may significantly aid in the management of FAW in the field by raising the likelihood of natural parasitism or predation [78] and forcing neonate larvae to feed on foliage with poor nutritional value to complete their life cycle, which reduces fecundity and survival [79]. However, the fluxametamide resistance development followed by the elevated activities of P450 and GST by the third instar FAW after successive fluxametamide selection could be contrasting with the fifth and sixth larval instars of Flux-SEL-(F10) generation. It could be attributed to the sensitivity of FAW against fluxametamide possibly varying among the larval instars after imposition of the selection pressure. The phenomenon of slowly increasing cantharidin resistance and P450 and GST activities in *Mythimna separata* after successive selection for 10 generations had a similar correlation with the development and fitness costs [80]. The fecundity and hatchability of FAW did not differ significantly in Flux-SEL (F5 and F10) generations, but the longevity of adult females and oviposition period were affected in the fluxametamide selection. Due to the suspension of feeding and a lower intake of food by the larval populations, we anticipated that the production of male sperm and viable female eggs, which are necessary for proper fertilization, was lower in the fluxametamide-selected FAW adults. Similar mechanisms have been linked to decreased adult longevity in *S. litura* [81]. Our results suggest that fluxametamide may have adverse effects on the reproductive physiology of FAW, even though the copulation and mating habits of adults that develop following fluxametamide selection pressure are unknown. Population parameters and life tables have been suggested as a more appropriate method for analyzing the overall impact of an insecticidal active ingredient on the population of an insect pest [82,83]. Moreover, it may be possible to predict more correctly how an insecticide will affect an insect population level by combining ecological and toxicological parameters. Therefore, to create potential resistance management solutions, it is crucial to understand how the high selection pressure of an insecticide affects the fitness costs of insecticide resistance [84,85]. The present study revealed that the *R_0_* and *T* were more significantly decreased in the Flux-SEL (F10) generation than in the susceptible (F0) population. This indicates the possibility of a slowdown in population dynamics of FAW after fluxametamide selection through a decrease in net reproductive rate and mean generation time. These findings are in contrast with the observation of Gope et al. [31], where a significant increase in *T* was noticed in the sublethal fluxametamide treatment in *P. xylostella*.

## 5. Conclusions

The findings of the resistance selection, cross-resistance pattern, detoxification enzyme activities, and life table analysis showed that there is very little chance that FAW will become highly resistant to fluxametamide. Yet, the data used to draw this conclusion came only from laboratory tests. Field conditions would be far more complicated and unpredictable. Fluxametamide resistance in FAW and other target pests in the field needs to be regularly monitored even if our findings suggested a low likelihood of its development. Further characterization of fluxametamide resistance in FAW in terms of target site mutation and genetic expression needs to be elucidated. In addition, a concrete relationship between novel chemicals such as fluxametamide or broflanilide and Bt toxin-expressing maize crop should be established for successful control of FAW and insecticide resistance management. Moreover, instead of using fluxametamide alone, insecticides for which cross-resistance has not been discovered could be tank-mixed with fluxametamide in an appropriate ratio in the sustainable management of FAW.

## Figures and Tables

**Table 1:** Toxicity of fluxametamide to different generations of *Spodoptera frugiperda* during the selection process.

Generation	Concentration for Selection (mg L^−1^)	N	LC_50_ (95% CI) (mg L^−1^)	Slope ± SE	ꭓ^2^ (df)	RF (95% CI)
Susceptible (F0)	-	280	0.024 (0.015–0.032)	1.56 ± 0.19	1.23 (5)	-
Flux-SEL (F1)	0.035	280	0.029 (0.021–0.036)	2.74 ± 0.32	1.85 (5)	1.21 (1.12–1.28)
Flux-SEL (F2)	0.043	280	0.025 (0.018–0.034)	2.49 ± 0.26	2.32 (5)	1.04 (0.93–1.13)
Flux-SEL (F3)	0.032	280	0.027 (0.021–0.034)	3.28 ± 0.18	3.51 (5)	1.13 (0.98–1.22)
Flux-SEL (F4)	0.037	280	0.032 (0.025–0.042)	2.67 ± 0.22	2.94 (5)	1.33 (1.19–1.44)
Flux-SEL (F5)	0.046	280	0.036 (0.024–0.049)	2.82 ± 0.29	1.77 (5)	1.50 (1.42–1.59)
Flux-SEL (F6)	0.051	280	0.045 (0.033–0.057)	3.08 ± 0.34	2.26 (5)	1.88 (1.76–1.99)
Flux-SEL (F7)	0.063	280	0.042 (0.029–0.053)	1.93 ± 0.21	2.47 (5)	1.75 (1.63–1.90)
Flux-SEL (F8)	0.058	280	0.049 (0.038–0.064)	2.51 ± 0.29	1.39 (5)	2.04 (1.89–2.17)
Flux-SEL (F9)	0.074	280	0.055 (0.043–0.069)	2.19 ± 0.25	3.15 (5)	2.29 (2.20–2.36)
Flux-SEL (F10)	0.096	280	0.063 (0.051–0.074)	2.75 ± 0.31	2.45 (5)	2.63 (2.52–2.74)

N: Total number of larvae used in bioassay; CI: Confidence Interval; RF (Resistance Factor) = LC_50_ of the fluxametamide-selected generation/LC_50_ of the susceptible F0 generation.

**Table 2:** Estimated realized heritability (*h*^2^) of resistance to fluxametamide in FAW.

Selected Generations	Response to Selection			Selection Differential				*h* ^2^
	Initial LC_50_ (95% CI)	Final LC_50_ (95% CI)	*R*	Average Slope	*σ_p_*	*i*	*S*	
10 (F0–F10)	0.024 (0.013–0.032)	0.063 (0.051–0.074)	0.042	2.548	0.393	1.273	0.501	0.084

**Table 3:** Cross-resistance pattern to eight tested insecticides in fluxametamide-selected F10 generation of *Spodoptera frugiperda*.

Insecticide	Generation	LC_50_ (95% CI) (mg L^−1^)	Slope ± SE	ꭓ^2^ (df)	RF (95% CI)
Broflanilide	Susceptible (F0)	0.025 (0.016–0.034)	1.62 ± 0.14	2.04 (5)	-
	Flux-SEL (F10)	0.029 (0.022–0.037)	1.49 ± 0.21	2.16 (5)	1.16 (0.88–1.45)
Chlorantraniliprole	Susceptible (F0)	2.268 (2.079–2.350)	2.31 ± 0.18	1.42 (5)	-
	Flux-SEL (F10)	3.231 (2.946–3.512)	1.79 ± 0.22	1.35 (5)	1.43 (0.97–1.75)
Emamectin benzoate	Susceptible (F0)	1.537 (1.414–1.628)	2.57 ± 0.29	1.98 (5)	-
	Flux-SEL (F10)	3.197 (2.993–3.345)	1.90 ± 0.15	2.12 (5)	2.08 (1.69–2.43)
Fipronil	Susceptible (F0)	5.193 (4.827–5.376)	1.49 ± 0.25	2.47 (5)	-
	Flux-SEL (F10)	6.803 (6.534–7.028)	1.88 ± 0.18	1.52 (5)	1.31 (0.92–1.67)
Indoxacarb	Susceptible (F0)	2.590 (2.380–2.764)	3.07 ± 0.20	1.70 (5)	-
	Flux-SEL (F10)	3.056 (2.892–3.174)	1.64 ± 0.19	1.32 (5)	1.18 (0.86–1.40)
Lambda cyhalothrin	Susceptible (F0)	3.946 (3.751–4.188)	1.81 ± 0.25	2.34 (5)	-
	Flux-SEL (F10)	4.893 (4.812–4.995)	2.39 ± 0.16	1.49 (5)	1.24 (0.95–1.52)
Spinetoram	Susceptible (F0)	0.039 (0.028–0.046)	1.63 ± 0.22	1.25 (5)	-
	Flux-SEL (F10)	0.041 (0.033–0.052)	1.56 ± 0.28	2.30 (5)	1.05 (0.71–1.36)
Tetraniliprole	Susceptible (F0)	0.028 (0.019–0.040)	1.45 ± 0.17	1.54 (5)	-
	Flux-SEL (F10)	0.039 (0.032–0.054)	1.96 ± 0.21	2.80 (5)	1.39 (0.98–1.75)

CI: Confidence Interval; RF (Resistance Factor) = LC_50_ of the Flux-SEL (F10) generation/LC_50_ of the susceptible F0 generation.

**Table 4:** Activities of detoxification enzymes in different generations of *Spodoptera frugiperda* selected with fluxametamide.

Generation	P450 (pmol min^−1^ mg pro^−1^)	Ratio	CarE (nmol min^−1^ mg pro^−1^)	Ratio	GST (μmol min^−1^ mg pro^−1^)	Ratio
Susceptible (F0)	0.83 ± 0.05 a	-	79.32 ± 1.21 a	-	12.47 ± 0.82 a	-
Flux-SEL (F5)	0.86 ± 0.08 a	1.04	84.16 ± 1.54 a	1.06	15.09 ± 1.04 ab	1.21
Flux-SEL (F10)	0.94 ± 0.07 ab	1.13	87.49 ± 1.67 a	1.10	24.18 ± 0.75 b	1.94

Values are means ± SE; Data in the same column followed by different alphabets are significantly different (*p* < 0.05); Ratio = Activity of the Flux-SEL generation/Activity of susceptible (F0) generation.

**Table 5:** Growth and development parameters of *Spodoptera frugiperda* in susceptible (F0) and fluxametamide-selected (F5 and F10) generations.

Generation	Developmental Duration (day)									
	Egg	1st Instar	2nd Instar	3rd Instar	4th Instar	5th Instar	6th Instar	Pupa	Adult Longevity	
									Male	Female
Susceptible (F0)	3.19 ± 0.03 a	2.09 ± 0.05 a	2.89 ± 0.04 a	3.07 ± 0.05 a	2.97 ± 0.04 a	3.16 ± 0.06 a	5.96 ± 0.05 a	12.35 ± 0.14 a	11.81 ± 0.18 a	12.42 ± 0.18 c
Flux-SEL (F5)	3.06 ± 0.02 a	2.13 ± 0.05 a	2.96 ± 0.03 a	2.99 ± 0.05 a	3.03 ± 0.03 a	3.19 ± 0.04 a	6.24 ± 0.07 ab	13.49 ± 0.09 b	11.64 ± 0.23 a	12.09 ± 0.21 b
Flux-SEL (F10)	3.12 ± 0.03 a	2.25 ± 0.06 b	2.93 ± 0.04 a	3.06 ± 0.03 a	3.12 ± 0.05 a	3.34 ± 0.06 b	6.78 ± 0.10 b	14.18 ± 0.12 b	11.39 ± 0.15 a	11.65 ± 0.13 a

Values are means ± SE; Data in the same column followed by different alphabets are significantly different (*p* < 0.05).

**Table 6:** Reproductive parameters of *Spodoptera frugiperda* in susceptible (F0) and fluxametamide-selected (F5 and F10) generations.

Generation	Reproductive Traits					
	Pre-Oviposition Period (day)	Oviposition Period (day)	Fecundity (Eggs Female^−1^)	Hatchability (%)	Pupation Rate (%)	Pupal Weight (g)
Susceptible (F0)	3.69 ± 0.18 a	5.28 ± 0.14 a	1296.17 ± 31.48 a	97.82 ± 0.67 a	82.14 ± 0.52 b	0.34 ± 0.04 b
Flux-SEL (F5)	4.05 ± 0.20 a	5.91 ± 0.08 a	1159.39 ± 47.26 a	90.19 ± 1.09 a	74.96 ± 0.78 ab	0.29 ± 0.04 b
Flux-SEL (F10)	3.87 ± 0.19 a	7.06 ± 0.11 b	1046.52 ± 29.07 a	85.45 ± 1.32 a	58.25 ± 1.15 a	0.21 ± 0.06 a

Values are means ± SE; Data in the same column followed by different alphabets are significantly different (*p* < 0.05).

**Table 7:** Life table parameters of susceptible (F0) and fluxametamide-selected (F5 and F10) generations of *Spodoptera frugiperda*.

Generation	Demographic Traits				
	*r* (day^−1^)	*λ* (day^−1^)	*R*_0_ (Offspring/Individual)	*T* (day)	*R_f_*
Susceptible (F0)	0.186 ± 0.01 a	1.235 ± 0.01 a	728.24 ± 54.31 b	35.434 ± 0.26 b	-
Flux-SEL (F5)	0.181 ± 0.01 a	1.230 ± 0.01 a	539.62 ± 76.25 b	34.756 ± 0.21 ab	0.741
Flux-SEL (F10)	0.175 ± 0.01 a	1.207 ± 0.01 a	257.14 ± 38.91 a	31.712 ± 0.33 a	0.353

Values are means ± SE; Different letters in a column indicate significant differences (*p* < 0.05) by a paired bootstrap test using the TWOSEX-MS chart program.

## Data Availability

The data presented in this study are available in Tables and Figures within the article or in the Supplementary Material provided.

## Supplementary Materials

The following supporting information can be downloaded at: https://www.mdpi.com/article/10.3390/toxics11040307/s1, Table S1: Collection details of *Spodoptera frugiperda* field populations from different sites in India.

## References

[1] Goergen G., Kumar P.L., Sankung S.B., Togola A., Tamò M. (2016). First report of outbreaks of the fall armyworm *Spodoptera frugiperda* (J. E. Smith) (Lepidoptera, Noctuidae), a new alien invasive pest in west and central Africa. PLoS ONE.

[2] Ganiger P.C., Yeshwanth H.M., Muralimohan K., Vinay N., Kumar A.R.V., Chandrashekara K. (2018). Occurrence of the new invasive pest, fall armyworm, *Spodoptera frugiperda* (J.E. Smith) (Lepidoptera: Noctuidae), in the maize fields of Karnataka, India. Curr. Sci..

[3] Early R., González-Moreno P., Murphy S.T., Day R. (2018). Forecasting the global extent of invasion of the cereal pest *Spodoptera frugiperda*, the fall armyworm. NeoBiota.

[4] Wu M., Qi G., Chen H., Ma J., Liu J., Jiang Y., Lee G., Otuka A., Hu G. (2021). Overseas immigration of fall armyworm, *Spodoptera frugiperda* (Lepidoptera: Noctuidae), invading Korea and Japan in 2019. Insect Sci..

[5] Kuate A.F., Hanna R., Fotio A.R.P.D., Abang A.F., Nanga S.N., Ngatat S., Tindo M., Masso C., Ndemah R., Suh C. (2019). *Spodoptera frugiperda* Smith (Lepidoptera: Noctuidae) in Cameroon: Case study on its distribution, damage, pesticide use, genetic differentiation and host plants. PLoS ONE.

[6] Sharanabasappa D., Kalleshwaraswamy C.M., Asokan R., Swamy H.M., Maruthi M.S., Pavithra H.B., Hegde K., Navi S., Prabhu S.T., Goergen G. (2018). First report of the fall armyworm, *Spodoptera frugiperda* (J E Smith) (Lepidoptera: Noctuidae), an alien invasive pest on maize in India. Pest Manag. Hort. Ecosyst..

[7] Sun X.X., Hu C.X., Jia H.R., Wu Q.L., Shen X.J., Zhao S.Y., Jiang Y.Y., Wu K.M. (2021). Case study on the first immigration of fall armyworm, *Spodoptera frugiperda* invading into China. J. Integr. Agric..

[8] Zhang D.D., Xiao Y.T., Xu P.J., Yang X.M., Wu Q.L., Wu K.M. (2021). Insecticide resistance monitoring for the invasive populations of fall armyworm, *Spodoptera frugiperda* in China. J. Integr. Agric..

[9] Kulye M., Mehlhorn S., Boaventura D., Godley N., Venkatesh S.K., Rudrappa T., Charan T., Rathi D., Nauen R. (2021). Baseline susceptibility of *Spodoptera frugiperda* populations collected in India towards different chemical classes of insecticides. Insects.

[10] Wu H.M., Feng H.L., Wang G.D., Zhang L.L., Zulu L., Liu Y.H., Zheng Y.L., Rao Q. (2022). Sublethal effects of three insecticides on development and reproduction of *Spodoptera frugiperda* (Lepidoptera: Noctuidae). Agronomy.

[11] Ahmad L., Habib Kanth R., Parvaze S., Sheraz Mahdi S., Ahmad L., Habib Kanth R., Parvaze S., Sheraz Mahdi S. (2017). Agro-climatic and agro-ecological zones of India. Experimental Agrometeorology: A Practical Manual.

[12] Firake D.M., Behere G.T. (2020). Natural mortality of invasive fall armyworm, *Spodoptera frugiperda* (J. E. Smith) (Lepidoptera: Noctuidae) in maize agroecosystems of northeast India. Biol. Control.

[13] Varshney R., Poornesha B., Raghavendra A., Lalitha Y., Apoorva V., Ramanujam B., Rangeshwaran R., Subaharan K., Shylesha A.N., Bakthavatsalam N. (2021). Biocontrol-based management of fall armyworm, *Spodoptera frugiperda* (J E Smith) (Lepidoptera: Noctuidae) on Indian Maize. J. Pl. Dis. Prot..

[14] Shylesha A.N., Jalali S.K., Gupta A., Varshney R., Venkatesan T., Shetty P., Ojha R., Ganiger P.C., Navik O., Subaharan K. (2018). Studies on new invasive pest *Spodoptera frugiperda* (J. E. Smith) (Lepidoptera: Noctuidae) and its natural enemies. J. Biol. Control.

[15] Roy D., Biswas S., Mondal D., Majumder S., Sarkar P.K. (2021). Efficacy and safety-evaluation of insecticidal modules against *Spodoptera frugiperda* (Lepidoptera: Noctuidae) and the residues of the most effective schedule in maize. Int. J. Trop. Insect Sci..

[16] Carvalho R.A., Omoto C., Field L.M., Williamson M.S., Bass C. (2013). Investigating the molecular mechanisms of organophosphate and pyrethroid resistance in the fall armyworm *Spodoptera frugiperda*. PLoS ONE.

[17] Bolzan A., Padovez F.E., Nascimento A.R., Kaiser I.S., Lira E.C., Amaral F.S., Kanno R.H., Malaquias J.B., Omoto C. (2019). Selection and characterization of the inheritance of resistance of *Spodoptera frugiperda* (Lepidoptera: Noctuidae) to chlorantraniliprole and cross-resistance to other diamide insecticides. Pest Manag. Sci..

[18] do Nascimento A.R.B., Farias J.R., Bernardi D., Horikoshi R.J., Omoto C. (2016). Genetic basis of *Spodoptera frugiperda* (Lepidoptera: Noctuidae) resistance to the chitin synthesis inhibitor lufenuron. Pest Manag. Sci..

[19] Okuma D.M., Bernardi D., Horikoshi R.J., Bernardi O., Silva A.P., Omoto C. (2018). Inheritance and fitness costs of *Spodoptera frugiperda* (Lepidoptera: Noctuidae) resistance to spinosad in Brazil. Pest Manag. Sci..

[20] Diez-Rodríguez G.I., Omoto C. (2001). Herança da resistência de *Spodoptera frugiperda* (J.E. Smith) (Lepidoptera: Noctuidae) a lambda-cialotrina. Neotrop. Entomol..

[21] Farias J.R., Andow D.A., Horikoshi R.J., Sorgatto R.J., Fresia P., dos Santos A.C., Omoto C. (2014). Field-evolved resistance to Cry1F maize by *Spodoptera frugiperda* (Lepidoptera: Noctuidae) in Brazil. Crop Prot..

[22] Horikoshi R.J., Bernardi D., Bernardi O., Malaquias J.B., Okuma D.M., Miraldo L.L., Amaral F.S., Omoto C. (2016). Effective dominance of resistance of *Spodoptera frugiperda* to Bt maize and cotton varieties: Implications for resistance management. Sci. Rep..

[23] Santos-Amaya O.F., Rodrigues J.V.C., Souza T.C., Tavares C.S., Campos S.O., Guedes R.N.C., Pereira E.J.G. (2015). Resistance to dual-gene Bt maize in *Spodoptera frugiperda*: Selection, inheritance and cross-resistance to other transgenic events. Sci. Rep..

[24] Boaventura D., Ulrich J., Lueke B., Bolzan A., Okuma D., Gutbrod O., Geibel S., Zeng Q., Dourado P.M., Martinelli S. (2020). Molecular characterization of Cry1F resistance in fall armyworm, *Spodoptera frugiperda* from Brazil. Insect Biochem. Mol. Biol..

[25] Sparks T.C., Storer N., Porter A., Slater R., Nauen R. (2021). Insecticide resistance management and industry: The origins and evolution of the Insecticide Resistance Action Committee (IRAC) and the mode of action classification scheme. Pest Manag. Sci..

[26] Asahi M., Kobayashi M., Kagami T., Nakahira K., Furukawa Y., Ozoe Y. (2018). Fluxametamide: A novel isoxazoline insecticide that acts via distinctive antagonism of insect ligand-gated chloride channels. Pestic. Biochem. Physiol..

[27] Umetsu N., Shirai Y. (2020). Development of the novel pesticides in the 21st century. J. Pestic. Sci..

[28] Kagami T., Hori M., Haruyama H. (2017). Studies on a novel insecticide, fluxametamide (Part 2) biological activity. Abstr. Annu. Meeting Pestic. Sci. Soc. Jpn..

[29] Mita T., Furukawa Y., Iwasa M., Kikuchi T., Komoda M., Maienfisch P., Mangelinckx S. (2021). Studies on a novel insecticide, fluxametamide. Recent Highlights in the Discovery and Optimization of Crop Protection Products.

[30] Jeschke P. (2021). Status and outlook for acaricide and insecticide discovery. Pest Manag. Sci..

[31] Gope A., Chakraborty G., Ghosh S.M., Sau S., Mondal K., Biswas A., Sarkar S., Sarkar P.K., Roy D. (2022). Toxicity and sublethal effects of fluxametamide on the key biological parameters and life history traits of diamondback moth *Plutella xylostella* (L.). Agronomy.

[32] Roy D., Sau S., Adhikary S., Biswas A., Biswas S., Chakraborty G., Sarkar P.K. (2022). Resistance risk assessment in diamondback moth, *Plutella xylostella* (L.) to fluxametamide. Crop Protec..

[33] Sayyed A.H., Omar D., Wright D.J. (2004). Genetics of spinosad resistance in a multi-resistant field-selected population of *Plutella xylostella*. Pest Manag. Sci..

[34] Lai T., Su J. (2011). Assessment of resistance risk in *Spodoptera exigua* (Hubner) (Lepidoptera: Noctuidae) to chlorantraniliprole. Pest Manag. Sci..

[35] Li Y., Wang Y., Qian C., Tang T., Shen N., Wu W., Wang J., Han Z., Zhao C. (2022). Lethal and sublethal effects of fluxametamide on rice-boring pest, rice stem borer *Chilo suppressalis*. Agronomy.

[36] Kumar N.T.D., Mohan K.M. (2022). Variations in the susceptibility of Indian populations of the fall armyworm, *Spodoptera frugiperda* (Lepidoptera: Noctuidae) to selected insecticides. Int. J. Trop. Insect Sci..

[37] Pinto J.R.L., Torres A.F., Truzi C.C., Vieira N.F., Vacari A.M., De Bortoli S.A. (2019). Artificial corn-based diet for rearing *Spodoptera frugiperda* (Lepidoptera: Noctuidae). J. Insect Sci..

[38] Gergs A., Baden C.U. (2021). A dynamic energy budget approach for the prediction of development times and variability in *Spodoptera frugiperda* rearing. Insects.

[39] Test Methods|Insecticide Resistance Action Committee (IRAC). https://irac-online.org/methods/.

[40] Tabashnik B.E. (1992). Resistance risk assessment: Realized heritability of resistance to *Bacillus thuringiensis* in diamondback moth (Lepidoptera: Plutellidae), tobacco budworm (Lepidoptera: Noctuidae), and Colorado potato beetle (Coleoptera: Chrysomelidae). J. Econ. Entomol..

[41] Tabashnik B.E., Mcgaughey W.H. (1994). Resistance risk assessment for single and multiple insecticides: Responses of Indian meal moth (Lepidoptera: Pyralidae) to *Bacillus thuringiensis*. J. Econ. Entomol..

[42] Chen C., Han P., Yan W., Wang S., Shi X., Zhou X., Desneux N., Gao X. (2018). Uptake of quercetin reduces larval sensitivity to lambda-cyhalothrin in *Helicoverpa armigera*. J. Pest Sci..

[43] Bradford M.M. (1976). A rapid and sensitive method for the quantitation of microgram quantities of protein utilizing the principle of protein-dye binding. Anal. Biochem..

[44] Nauen R., Stumpf N. (2002). Fluorometric microplate assay to measure glutathione *S*-transferase activity in insects and mites using monochlorobimane. Anal. Biochem..

[45] Grant D.F., Bender D.M., Hammock B.D. (1989). Quantitative kinetic assays for glutathione *S*-transferase and general esterase in individual mosquitoes using an EIA reader. Insect Biochem..

[46] Abbott S.W. (1925). A method of computing the effectiveness of an insecticide. J. Econ. Entomol..

[47] Robertson J.L., Russell R.M., Preisler H.K., Savin N.E. (2007). Pesticide Bioassays with Arthropods.

[48] Chi H., Liu H.I.S. (1985). Two new methods for the study of insect population ecology. Bull. Inst. Zool. Acad. Sin..

[49] Chi H. (1988). Life-table analysis incorporating both sexes and variable development rates among individuals. Environ. Entomol..

[50] Chi H. (2018). TWOSEX-MSChart: A Computer Program for the Age Stage, Two-Sex Life Table Analysis. http://140.120.197.173/Ecology/Download/Twosex-MSChart-exe-B200000.rar.

[51] Moreno R.G., Sanchez D.M., Blanco C.A., Whalon M.E., Santofimio H.T., Maciel J.C.R., DiFonzo C. (2019). Field-evolved resistance of the fall armyworm (Lepidoptera: Noctuidae) to synthetic insecticides in Puerto Rico and Mexico. J. Econ. Entomol..

[52] Sparks T.C., Crossthwaite A.J., Nauen R., Banba S., Cordova D., Earley F., Ebbinghaus-Kintscher U., Fujioka S., Hirao A., Karmon D. (2020). Insecticides, biologics and nematicides: Updates to IRAC’s mode of action classification—A tool for resistance management. Pestic. Biochem. Physiol..

[53] Yainna S., Negre N., Silvie P.J., Brevault T., Tay W.T., Gordon K., dAlencon E., Walsh T., Nam K. (2021). Geographic monitoring of insecticide resistance mutations in native and invasive populations of the fall armyworm. Insects.

[54] Jia Z.Q., Zhan E.L., Zhang S.G., Jones A.K., Zhu L., Wang Y.N., Huang Q.T., Han Z.J., Zhao C.Q. (2022). Sublethal doses of broflanilide prevents molting in the fall armyworm, *Spodoptera frugiperda* via altering molting hormone biosynthesis. Pestic. Biochem. Physiol..

[55] Ijaz M., Afzal M.B.S., Shad S.A. (2016). Resistance risk analysis to acetamiprid and other insecticides in acetamiprid-selected population of *Phenacoccus solenopsis*. Phytoparasitica.

[56] Ismail M., Ejaz M., Abbas N., Shad S.A., Afzal M.B.S. (2017). Resistance risk assessment to chlorpyrifos and cross-resistance to other insecticides in a field strain of *Phenacoccus solenopsis* Tinsley. Crop Protect..

[57] Abbas N., Shad S.A. (2015). Assessment of resistance risk to lambda-cyhalothrin and cross-resistance to four other insecticides in the house fly, *Musca domestica* L. (Diptera: Muscidae). Parasitol. Res..

[58] Mousseau T.A., Roff D.A. (1987). Natural selection and heritability of fitness components. Heredity.

[59] Diez J.D.R., Benjumea C.I.S. (2011). Susceptibility of *Spodoptera frugiperda* (Lepidoptera: Noctuidae) strains from central Colombia to two insecticides, methomyl and lambda cyhalothrin: A study of the genetic basis of resistance. J. Econ. Entomol..

[60] Gorman K., Slater R., Blande J.D., Clarke A., Wren J., McCaffery A., Denholm I. (2010). Cross-resistance relationships between neonicotinoids and pymetrozine in *Bemisia tabaci* (Hemiptera: Aleyrodidae). Pest Manag. Sci..

[61] Elbert A., Nauen R. (2000). Resistance of *Bemisia tabaci* (Homoptera: Aleyrodidae) to insecticides in southern Spain with special reference to neonicotinoids. Pest Manag. Sci..

[62] Mahalanobish D., Dutta S., Roy D., Biswas A., Sarkar S., Mondal D., Gaber A., Hossain A., Sarkar P.K. (2022). Field-evolved resistance and mechanisms in *Bemisia tabaci* Asia I to a novel pyropene insecticide, afidopyropen, in India. Crop Protect..

[63] Bernardi D., Salmeron E., Horikoshi R.J., Bernardi O., Dourado P.M., Carvalho R.A., Martinelli S., Head G.P., Omoto C. (2015). Cross-resistance between Cry1 proteins in fall armyworm (*Spodoptera frugiperda*) may affect the durability of current pyramided Bt maize hybrids in Brazil. PLoS ONE.

[64] Lira E.C., Bolzan A., Nascimento A.R.B., Amaral F.S.A., Kanno R.H., Kaiser I.S., Omoto C. (2020). Resistance of *Spodoptera frugiperda* (Lepidoptera: Noctuidae) to spinetoram: Inheritance and cross-resistance to spinosad. Pest Manag. Sci..

[65] Hafeez M., Li X., Ullah F., Zhang Z., Zhang J., Huang J., Chen L., Siddiqui J.A., Ren X., Zhou S. (2022). Characterization of indoxacarb resistance in the fall armyworm: Selection, inheritance, cross-resistance, possible biochemical mechanisms, and fitness costs. Biology.

[66] Sun X.I., Wei R., Li L., Zhu B., Liang P., Gao X. (2021). Resistance and fitness costs in diamondback moths after selection using broflanilide, a novel meta-diamide insecticide. Insect Sci..

[67] Roy D., Samanta A., Biswas A., Chakraborty G., Sarkar P.K. (2021). Insecticide resistance status of *Hyposidra talaca* (Lepidoptera: Geometridae) in major tea growing zone of India. Phytoparasitica.

[68] Giraudo M., Hilliou F., Fricaux T., Audant P., Feyereisen R., LeGoff G. (2015). Cytochrome P450s from the fall armyworm (*Spodoptera frugiperda*): Responses to plant allelochemicals and pesticides. Insect Mol. Biol..

[69] Pu X., Yang Y., Wu S., Wu Y. (2010). Characterization of abamectin resistance in a field-evolved multiresistant population of *Plutella xylostella*. Pest Manag. Sci..

[70] Gong Y.J., Wang Z.H., Shi B.C., Kang Z.J., Zhu L., Jin G.H., Wei S.J. (2013). Correlation between pesticide resistance and enzyme activity in the diamondback moth, *Plutella xylostella*. J. Insect Sci..

[71] Punzo F. (1993). Detoxification enzymes and the effect of temperature on the toxicity of pyrethroids to the fall armyworm, *Spodoptera frugiperda* (Lepidoptera: Noctuidae). Comp. Biochem. Physiol..

[72] Saingamsook J., Yanola J., Lumjuan N., Walton C., Somboon P. (2019). Investigation of relative development and reproductivity fitness cost in three insecticide-resistant strains of *Aedes aegypti* from Thailand. Insects.

[73] Kliot A., Ghanim M. (2012). Fitness costs associated with insecticide resistance. Pest Manag. Sci..

[74] Barbosa M.G., Andre T.P.P., Pontes A.D.S., Souza S.A., Oliveira N.R.X., Pastori P.L. (2020). Insecticide rotation and adaptive fitness cost underlying insecticide resistance management for *Spodoptera frugiperda* (Lepidoptera: Noctuidae). Neotrop. Entomol..

[75] Santos-Amaya O.F., Tavares C.S., Rodrigues J.V.C., Oliveira E.E., Guedes R.N.C., Pereira E.J.G. (2022). Strong fitness costs of fall armyworm resistance to dual-gene Bt maize are magnified on less-suitable host-crop cultivars. Agronomy.

[76] Hannig G.T., Ziegler M., Marcon P.G. (2009). Feeding cessation effects of chlorantraniliprole, a new anthranilic diamide insecticide, in comparison with several insecticides in distinct chemical classes and mode-of-action groups. Pest Manag. Sci..

[77] Liu D., Jia Z.Q., Peng Y.C., Sheng C.W., Tang T., Xu L., Han Z.J., Zhao C.Q. (2018). Toxicity and sublethal effects of fluralaner on *Spodoptera litura* Fabricius (Lepidoptera: Noctuidae). Pestic. Biochem. Physiol..

[78] Weseloh R. (1984). Effects of the feeding inhibitor plictran and low *Bacillus thuringiensis* Berliner doses on *Lymantria dispar* (L.) (Lepidoptera: Lymantriidae): Implications for *Cotesia melanoscelus* (Ratzeburg) (Hymenoptera: Braconidae). Environ. Entomol..

[79] Erb S.L., Bourchier R.S., van Frankenhuyzen K., Smith S.M. (2001). Sublethal effects of *Bacillus thuringiensis* Berliner subsp. kurstaki on *Lymantria dispar* (Lepidoptera: Lymantriidae) and the tachinid parasitoid *Compsilura concinnata* (Diptera: Tachinidae). Environ. Entomol..

[80] Li Y., Sun H., Yasoob H., Tian Z., Li Y., Li R., Zheng S., Liu J., Zhang Y. (2021). Biogenetic cantharidin is a promising leading compound to manage insecticide resistance of *Mythimna separata* (Lepidoptera: Noctuidae). Pestic. Biochem. Physiol..

[81] Shahout H.A., Xu J.X., Yao X.M., Jia Q.D. (2011). Influence and mechanism of different host plants on the growth, development, and fecundity of reproductive system of common cut worm *Spodoptera litura* (Fabricius) (Lepidoptera: Noctuidae). Asian J. Agric. Sci..

[82] Stark J.D., Banks J.E. (2003). Population-level effects of pesticides and other toxicants on arthropods. Annu. Rev. Entomol..

[83] Hamedi N., Fathipour Y., Saber M. (2010). Sublethal effects of fenpyroximate on life table parameters of the predatory mite *Phytoseius plumifer*. BioControl.

[84] Yu N., Tian J., Zhang Y., Li Z., Liu Z. (2018). Imidacloprid-susceptible *Nilaparvata lugens* individuals exceeded resistant individuals in a mixture population with density pressure. Pest Manag. Sci..

[85] Wang R., Qu C., Wang Z., Yang G.F. (2020). Cross-resistance, biochemical mechanism and fitness costs of laboratory-selected resistance to pyridalyl in diamondback moth, *Plutella xylostella*. Pestic. Biochem. Physiol..

