# Supplementary material for: Risk Assessment of Fluxametamide Resistance and Fitness Costs in Fall Armyworm (Spodoptera frugiperda)
Source: Toxics. 2023 Mar 26;11(4):307. doi: 10.3390/toxics11040307 (PMC10144201; doi:10.3390/toxics11040307)
Supplement: Supplementary file 1 [file toxics-11-00307-s001.zip › toxics-2276260-supplementary.pdf]

Table S1: Collection details of *Spodoptera frugiperda* field populations from different sites in India.

Name of the Province	District	Latitude	Longitude	Crop
West Bengal	Murshidabad	24.1759 ^{0}N	88.2802 ^{0}E	Maize
	Malda	25.0108 ^{0}N	88.1411 ^{0}E	Maize
	North Dinajpur	25.9810 ^{0}N	88.0510 ^{0}E	Maize
Bihar	Bhojpur	25.4662 ^{0}N	84.5222 ^{0}E	Maize
Madhya Pradesh	Indore	22.7196 ^{0}N	75.8577 ^{0}E	Maize
Andhra Pradesh	Prakasam	15.3485 ^{0}N	79.5603 ^{0}E	Maize
